# Attachment ability of the polyphagous bug *Nezara viridula* (Heteroptera: Pentatomidae) to different host plant surfaces

**DOI:** 10.1038/s41598-018-29175-2

**Published:** 2018-07-20

**Authors:** Gianandrea Salerno, Manuela Rebora, Elena Gorb, Stanislav Gorb

**Affiliations:** 1 Dipartimento di Scienze Agrarie, Alimentari e Ambientali, University of Perugia, Borgo XX Giugno, Perugia, Italy; 2 Dipartimento di Chimica, Biologia e Biotecnologie, University of Perugia, Via Elce di Sotto 8, 06121 Perugia, Italy; 3 Department of Functional Morphology and Biomechanics, Zoological Institute, Kiel University, Am Botanischen Garten 9, 24098 Kiel, Germany

## Abstract

The present investigation tests through friction experiments the attachment ability of adults of the southern green stink bug *Nezara viridula* L. (Heteroptera: Pentatomidae), a polyphagous insect representing a cosmopolitan pest, on different host plant species characterized by smooth, hairy and waxy surfaces. Surfaces of different tested plants have been studied in Cryo-Scanning Electron Microscope (Cryo-SEM). The load cell force transducer was used to evaluate the potential damage to the insect attachment devices induced by walking on the different leaf surfaces. In case of the plant *Phaseolus vulgaris*, where insects showed a strong reduction in their adhesion ability during and after walking on the leaf, the damage to the insect by two cultivars with different morphological features and the insect ability to recover after 24 h has been evaluated. The ability to recover notwithstanding the damage to attachment devices, shown by Cryo-SEM investigations, together with the strong attachment forces produced on various plant leaves, characterized by different morphological features, is in agreement with the great adaptability and ecological plasticity of this widely-spread bug species. The present study, increasing our knowledge on the mechanical interaction of this species with different host plant species, can help to develop new strategies to control this insect pest.

## Introduction

Plants and herbivorous insects are united by intricate relationships. During the long period of coevolution between insects and plants, these last developed a wide diversity of features, not only to attract pollinators, but also to defense against herbivores. These features are chemical and physical barriers affecting insect performance on the plant surface. In this context, the evolution of plant surfaces and insect attachment pads is an interesting example of competition between insect attachment systems and plant anti-attachment surfaces^1^. Many plants have impenetrable barriers, such as specialized bark and cristalline wax covered cuticles or other surface adaptations, such as thorns and spines, to protect them from herbivores (see review in^2^). Insects have to cope with these adaptations by further development of their attachment systems enabling them to adhere to their host plants. Plant glandular trichomes may exude sticky or poisonous chemicals, while non-glandular trichomes, which do not secrete such chemicals, may on the other hand impale, entangle or impede locomotion of insects by disturbing physical interactions between insects and plants^3,4^. The chemical ecology of insect-plant interactions was widely studied in the last decades, but relatively few data are available regarding the mechanical interactions between insects and plants, especially considering that the attachment ability to various plant substrates is a crucial factor for the evolutionary success of herbivore insects^4^.

To achieve sufficient attachment for locomotion on widely diverse plant surfaces, insects evolved various types of leg attachment devices^5^. Claws are adapted to interlock themselves with rough surfaces^6–8^, when the distances between adjacent asperities, as well as their heights are larger than the claw tip diameter^9^. On smooth surfaces, insects attach themselves using their tarsal adhesive devices, such as hairy pads (for example flies, beetles, earwigs), or smooth flexible pads called arolia, pulvilli and euplantulae (for example cockroaches, ants, aphids, grasshoppers, bugs, butterflies, moths)^5,10,11^. In general, insect adhesive pads are flexible areas of cuticle supplemented with cuticular secretions, whose presence has been demonstrated in both alternative designs of the pads, hairy and smooth (see review in^12^).

To date, several studies have been carried out to characterize the attachment systems of different insect taxa including Blattodea, Coleoptera, Diptera, Hymenoptera, Orthoptera, Thysanoptera, Homoptera and Heteroptera (for a detailed bibliography see introduction in^13^). In this last suborder, experimental studies on the attachment devices are limited to one species of Coreidae^14^ and two of Miridae^8,15–17^. Regarding Pentatomidae, a family encompassing numerous dangerous pest species of agricultural importance, few data are available in particular on the southern green stink bug *Nezara viridula* L. (Heteroptera: Pentatomidae)^13,18^. This pentatomid species is a serious cosmopolitan pest of more than 30 different crops in most areas of the world^19,20^. It attacks plants belonging to at least 32 families^20^.

Recent investigations allowed to characterize the attachment ability of males and females of *N*. *viridula* on hydrophilic and hydrophobic artificial surfaces and on the adaxial and abaxial leaf surfaces of the model plant species *Vicia faba*^13^. In another study, bug attachment ability was tested with similar technique on few other plant species^18^. Moreover, friction of *N*. *viridula* was evaluated on artificial substrates with different roughness^13^. Ultrastructural investigations (Cryo-SEM, TEM and confocal laser scanning microscopy (CLSM)) described in detail the pretarsus of *N*. *viridula* bearing claws, smooth flexible pads (pulvilli) and hairy pads on the ventral side of the basitarsus (Supplementary Fig. S1). No sexual dimorphism has been revealed in morphology of attachment devices at different levels of the structural organisation^21^. To evaluate the role of these attachment devices, behavioural experiments testing *N*. *viridula* with ablated pulvilli, hairs and claws, using a traction force experiments set up, have been performed on artificial substrates characterised by different roughness and on substrates with different surface energies and underwater^22^.

The aim of the present investigation is to deepen the knowledge on the attachment ability of the green stinkbug *N*. *viridula* at the adult stage to different host plant surfaces, in order to understand the adaptability of a polyphagous insect to plants with different leaf surfaces. The differences in its attachment ability on different host plant species, characterized by smooth, hairy and waxy surfaces has been evaluated through friction experiments on tethered insects using a load cell force transducer. Surfaces of different tested plants were studied in detail under Cryo-Scanning Electron Microscope (Cryo-SEM). Furthermore, the load cell force transducer has been used, to evaluate in *N*. *viridula* adults the potential damage of insect attachment devices induced by walking on the different leaf surfaces. In case of the plant *Phaseolus vulgaris*, where insects showed a strong reduction in their adhesion ability after walking on the leaf, the damage to the insect by two cultivars with different morphological features and the insect ability to recover has been evaluated.

## Results

### Plant surface characterisation

**Figure 1:**
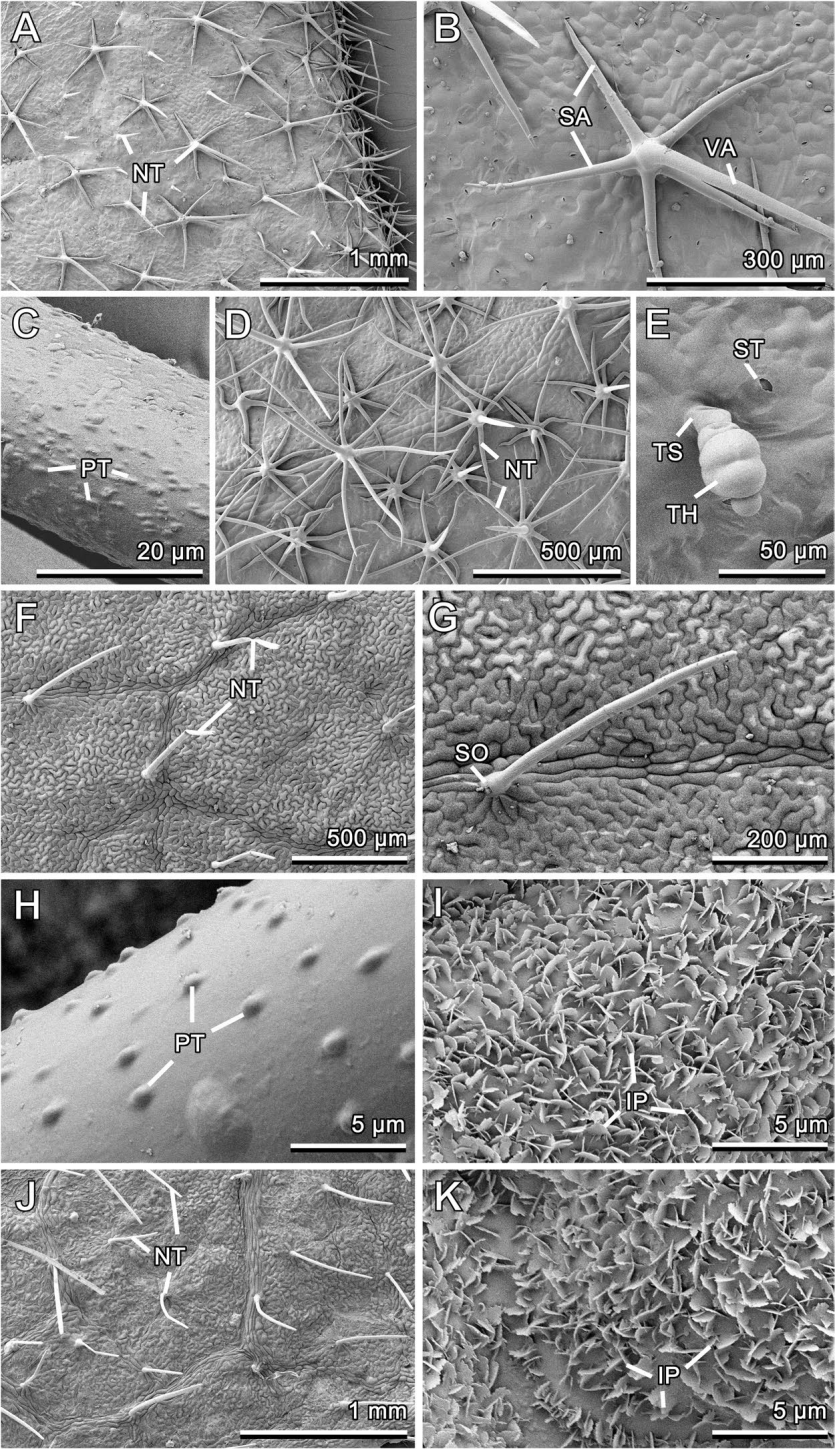
Cryo-SEM micrographs of the adaxial (**A**–**C**) and abaxial (**D**,**E**) leaf surfaces in *Solanum melongena* and of the adaxial (**F**–**I**) and abaxial (**J**,**K**) leaf surfaces in *Glycine max*. (**A**,**D**,**F**,**J**) General views showing non-glandular trichomes. (**B**,**G**) Non-glandular trichome. (**C**,**H**) Surface of the non-glandular trichome. (**E**) Glandular trichome. (**I**,**K**) 3D epicuticular waxes on the adaxial and abaxial leaf sides, respectively. IP, irregular wax platelet; NT, non-glandular trichome; PT, protrusion; SA, side arm; SO, socket; ST, stoma; TH, glandular trichome head; TS; trichome stalk; VA, vertical arm.

The surface architecture on both sides of *Solanum melongena* leaves is characterized by a dense pubescence formed by non-glandular stellate trichomes (Fig. 1A and 1D). These multicellular structures bear one vertical arm and from 2 to 8 (more often >5) spread accumbent side arms (Fig. 1B). On the adaxial leaf side, also trichomes with only the vertical arm are present (Fig. 1A). The arm length varies greatly within the same trichome and between different trichomes. Spread arms on the adaxial leaf side are shorter (200.21 ± 105.55 μm, N = 20) and in significantly lesser number (4.29 ± 0.88 trichome^−1^, N = 23) than those on the abaxial side (length: 269.13 ± 107.36 μm, N = 25; number: 7.39 ± 0.82 trichome^−1^, N = 18)) (compare Fig. 1A,D). Moreover, on the abaxial leaf side, spread arms build a multi-layer coverage (Fig. 1D) due to, among others, much higher trichome density: ca. 17 mm^−2^ here vs ca. 5 mm^−2^ on the adaxial side. The trichome surface is rather rough at the microscale level because of nodose knobby irregular outgrowths (Fig. 1C); this was especially well pronounced on the adaxial leaf side. Also relatively small (length: 61.21 ± 19.51 μm, N = 4) glandular capitate trichomes with rather short stalks and ellipsoid multicellular heads (length ratio stalk to head is about 1:1) (Fig. 1E) are solitary scattered (density: < 1 per 1 mm^−2^) over both leaf surfaces. The surface underneath trichomes is smooth, slightly uneven, with numerous stomata on both leaf sides (density is about 150 mm^−2^) (Fig. 1B,E).

In *Glycine max*, the surface texture of both leaf sides is formed by slightly convex (especially distinct on the abaxial side) shape of epidermal cells bearing both non-grandular trichomes and 3D epicuticular wax coverage (Fig. 1F,I,J,K). Surface micromorphology is very similar in both leaf sides, although Lin and Hu^23^ found epicuticular waxes only on the abaxial leaf surface. Unicellular, non-branched, nearly finger-shaped long (length: 381.60 ± 94.17 μm, N = 17) and thin trichomes with prominent sockets have slightly inflated bases and obtuse tips (Fig. 1G). They cover leaf surfaces very uniformly, however, tree-fold more densely the abaxial leaf side (density is about 3 mm^−2^ (adaxial) and 9 mm^−2^ (abaxial)) (compare 1F and 1J). These slightly inclined trichomes readily collapse either by bending in the middle/apical region or become flat (Fig. 1F). The trichome surface is microsculptured with numerous prominent half-spherical/half-ellipsoid nodose knobby protrusions (Fig. 1H). On both leaf sides, the surface underneath the indumentum bears very uniform, regular and dense (ca. 4–5 projections per 1 μm^2^) wax coverage composed of one layer of irregular platelets (Fig. 1I,K). These microscopic (length: 1.12 ± 0.25 μm, N = 30; width: 0.51 ± 0.09 μm, N = 20) flat wax projections with irregular margin protrude more or less perpendicularly from the surface. Contrary to previous authors indicating either asterisk wax projections^23^ or rosettes composed of five to ten platelets^24^, we detected rather low degree of aggregation of irregular wax platelets. Stomata occurring on both leaf sides (density on the abaxial side is about 80 mm^−2^) are poorly visible on the adaxial surface.

**Figure 2:**
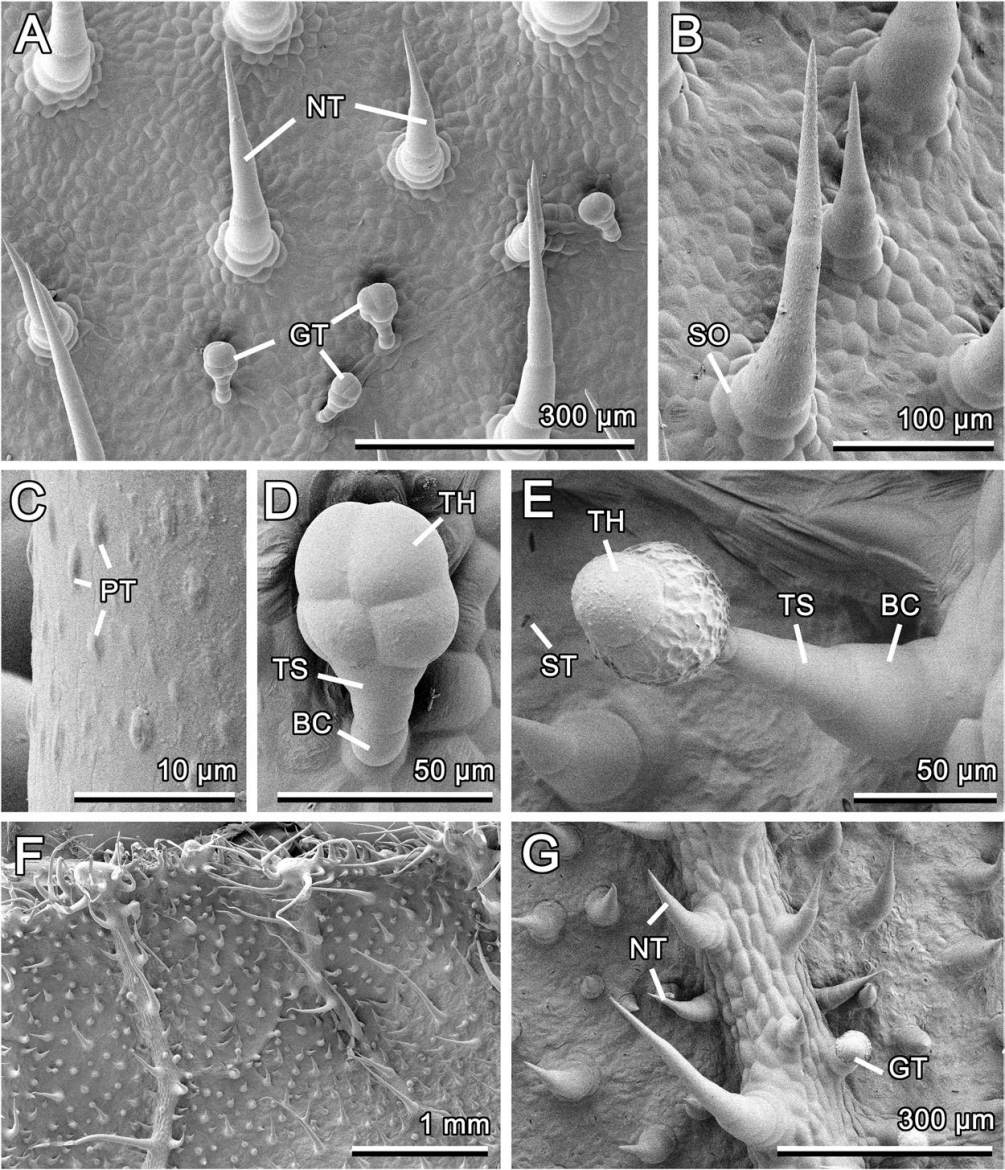
Cryo-SEM micrographs of the adaxial (**A**–**D**) and abaxial (**E**–**G**) leaf surfaces in *Cucurbita pepo*. (**A**,**F,G**) General views showing different surface features. (**B**) Non-glandular trichomes. (**C**) Surface of the non-glandular trichome. (**D**,**E**) Glandular trichomes from the adaxial and abaxial leaf sides, respectively. BC, basal cell; GT, glandular trichome; NT, non-glandular trichome; PT, protrusion; SO, socket; ST, stoma; TH, glandular trichome head; TS; trichome stalk.

The adaxial and abaxial surfaces of *Cucurbita pepo* leaf show numerous non-glandular and glandular trichomes (Fig. 2A,F,G). Non-glandular trichomes regularly cover the areas between the leaf veins (both leaf sides) and on the veins (the abaxial side), whereas glandular ones are almost completely associated with the veins on both leaf sides. Non-glandular trichomes on both leaf surfaces belong to the same type. They are multicellular, uniseriate, with multicellular sockets (Fig. 2B). These trichomes are non-branched, cone-shaped, with sharp tips. The trichome surface bears a very noticeable sculpturing, due to plentiful microscopic half-spherical/half-ellipsoid nodose knobby protrusions (Fig. 2C). On the adaxial side, these slightly inclined/bent trichomes, having a greatly variable length (0.09–1.02 mm; 313.61 ± 236.33 μm, N = 20), are responsible for the uniform and dense (ca. 13–23 trichomes per 1 mm^2^) pubescence. Glandular trichomes (total length: 63.98 ± 4.96 μm, N = 10) of the adaxial side, each containing a basal cell, uniseriate two-celled stalk (summed length: 26.04 ± 3.03 μm, N = 10) and four-celled head (length: 37.29 ± 4.12 μm, N = 10) (Fig. 2D), are well-described short-stalked *Cucurbita* trichomes previously found on both leaf sides of *C*. *pepo* subsp. *pepo* var. *styriaca* (type I according to^25,26^). These trichomes cover the surface more sparsely (ca. 7 per 1 mm^2^) than non-glandular ones.

On the abaxial side, long non-glandular trichomes (length: 596.88 ± 163.23 μm, N = 20) are more frequent on the leaf veins, which are very prominent here, whereas short ones (length: 130.34 ± 56.01 μm, N = 30) are present everywhere (Fig. 2F,G). The indumentum formed by non-glandular trichomes is more than twice as dense (ca. 35 trichomes per 1 mm^2^) as that on the adaxial leaf side. We did not find large multicellular barrel-shaped trichomes detected previously by Popa and Şipoş^27^ in *C*. *pepo*. Glandular trichomes differ essentially from those of the adaxial side. These long-stalked trichomes (total length: 132.72 ± 4.96 μm, N = 10) correspond to the type II (‘neck-cell’ type) described on both leaf sides of *C*. *pepo* subsp. *pepo* var. *styriaca*^25,26^. They are characterized by the two-celled head region (length: 52.85 ± 7.32 μm, N = 3), distinctly separated from the multicellular uniseriate stalk, and one basal cell (summed length: 79.02 ± 15.53 μm (N = 3)) (Fig. 2E). The density of the glandular trichome coverage is nearly 2 mm^−2^ being essentially lower than that on the adaxial side. Numerous stomata (density: > 200 per 1 mm^2^) are regularly scattered between trichomes only on the abaxial leaf side (Fig. 2E).

**Figure 3:**
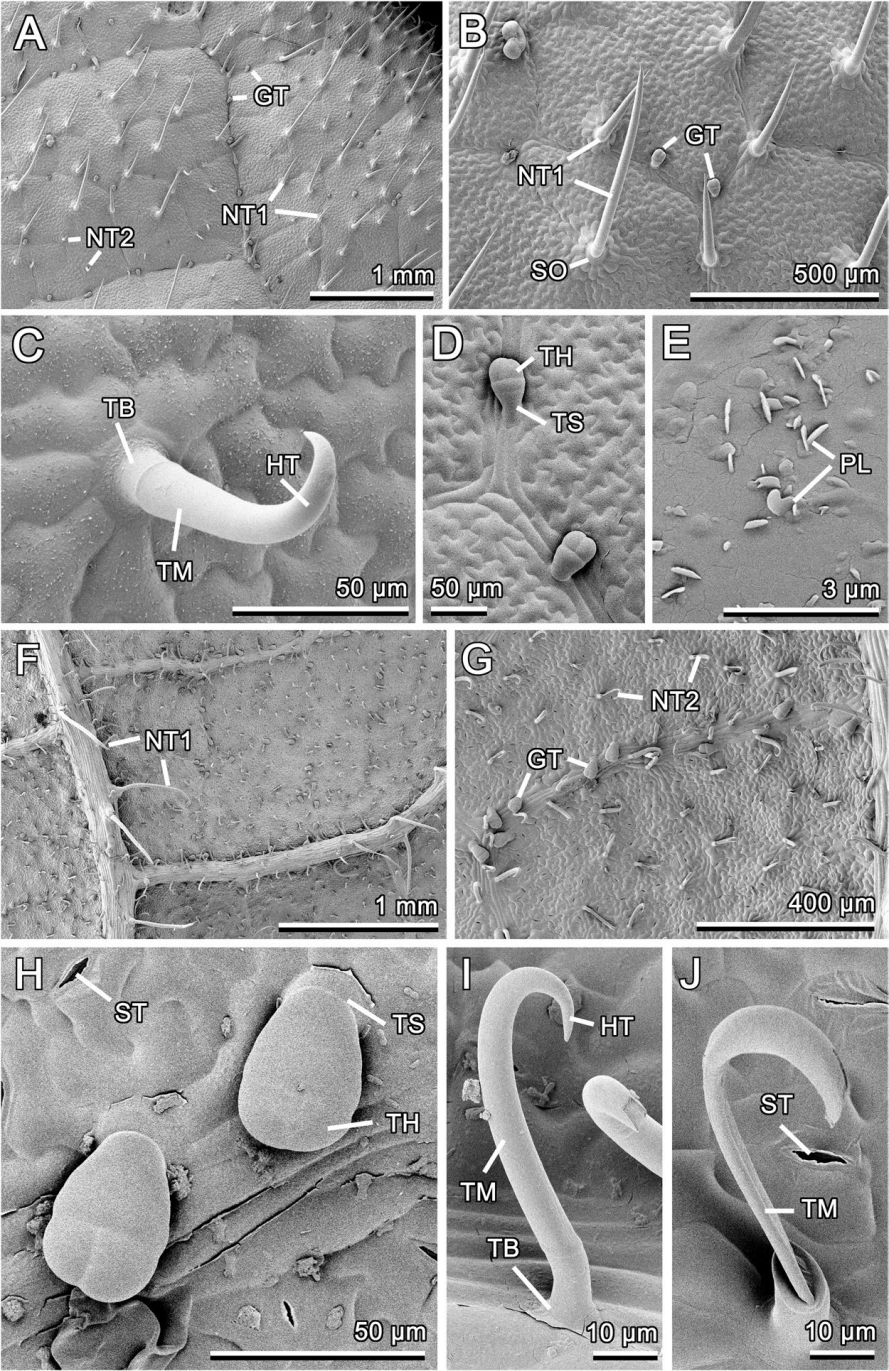
Cryo-SEM micrographs of the adaxial (**A**–**E**) and abaxial (**F**–**J**) leaf surface in *Phaseolus vulgaris*. (**A**,**B**,**F**,**G**) General views showing different surface features. (**C**,**I**,**J**) Non-glandular trichome of the second type: intact (**C**,**I**) and collapsed (**J**). (**D**,**H**) Glandular trichomes. (**E**) 3D epicuticular wax. GT, glandular trichome; HT, hook-shaped tip of the NT2; NT1, non-glandular trichome of the first type; NT2, non-glandular trichome of the second type; PL, wax platelet; SO, socket; ST, stoma; TB, base of the NT2; TM, middle part of the NT2; TH, glandular trichome head; TS; trichome stalk.

Both leaf surfaces in *Phaseolus vulgaris* bear all three types (two non-glandular and one glandular) of non-branched trichomes, but their density and distribution differ between the adaxial and abaxial sides (Fig. 3A,B,F,G). The first type is presented by dart-shaped non-glandular trichomes with well developed multicellular socket and very sharp tip (Fig. 3B). On the adaxial leaf side, these trichomes, which vary greatly in size (length: 259.12 ± 155.76 μm, N = 30), protrude at very shallow angles and are pointed to one preferred direction (Fig. 3A,B). They are responsible for a regular and dense (density: ca. 10 mm^−2^) anisotropic coverage on the surface (Fig. 3A). On the abaxial side, such trichomes are slightly longer and more uniform in size (length: 352.96 ± 106.12 μm, N = 8), have more upright position, do not show specific orientation, and occur in a small number (ca. 1–2 mm^−2^) only on main, very prominent leaf veins (Fig. 3F). We did not observe straight short pen- like trichomes described previously by Gepp^28^ on the adaxial leaf side in *P*. *vulgaris*. The second type of non-glandular trichomes includes characteristic hook-shaped structures having small multicellular sockets and sharp tips pointed to different directions. In these trichomes, several regions are clearly seen (Fig. 3C,I): (1) very stable, presumably solid basal part, (2) flexible middle part, which is hollow inside and readily collapses becoming flat or crumpled (Fig. 3J), and (3) stable solid hooked part. They are relatively small (height: 61.48 ± 7.48 μm, N = 9) and sparsely (max. 5 mm^−2^) distributed on or in the vicinity of veins on the adaxial leaf side (Fig. 3A). On the abaxial side, these trichomes are much larger (height: 188.89 ± 50.82 μm, N = 19), especially on veins, and very densely (density: ca. 100 mm^−2^) cover the surface everywhere (Fig. 3F,G). Glandular trichomes (the third trichome type) are rather small (length: 59.13 ± 25.95 μm, N = 8), clavate, with very short stalks and relatively large, four-celled, accumbent glandular heads (length ratio stalk to head is about 1:8) (Fig. 3D,H). These trichomes are mostly associated with leaf veins and occur on both leaf surfaces, however, more densely on the abaxial side: ca. 5 vs 7 mm^−2^ on the adaxial and abaxial sides, respectively. The adaxial leaf surface additionally bears very loose and inhomogeneously distributed 3D epicuticular wax coverage formed by microscopic (length: 0.61 ± 0.05 μm, N = 14; height: 0.32 ± 0.17 μm, N = 5; thickness: 0.06 ± 0.01 μm, N = 10) flat platelets with entire margin (Fig. 3E). The platelets are often semicircular in shape and most of them protrude perpendicularly from the surface. Numerous stomata (on an average ca. 180 mm^−2^) are present only on the abaxial leaf surface (Fig. 3H,J).

**Figure 4:**
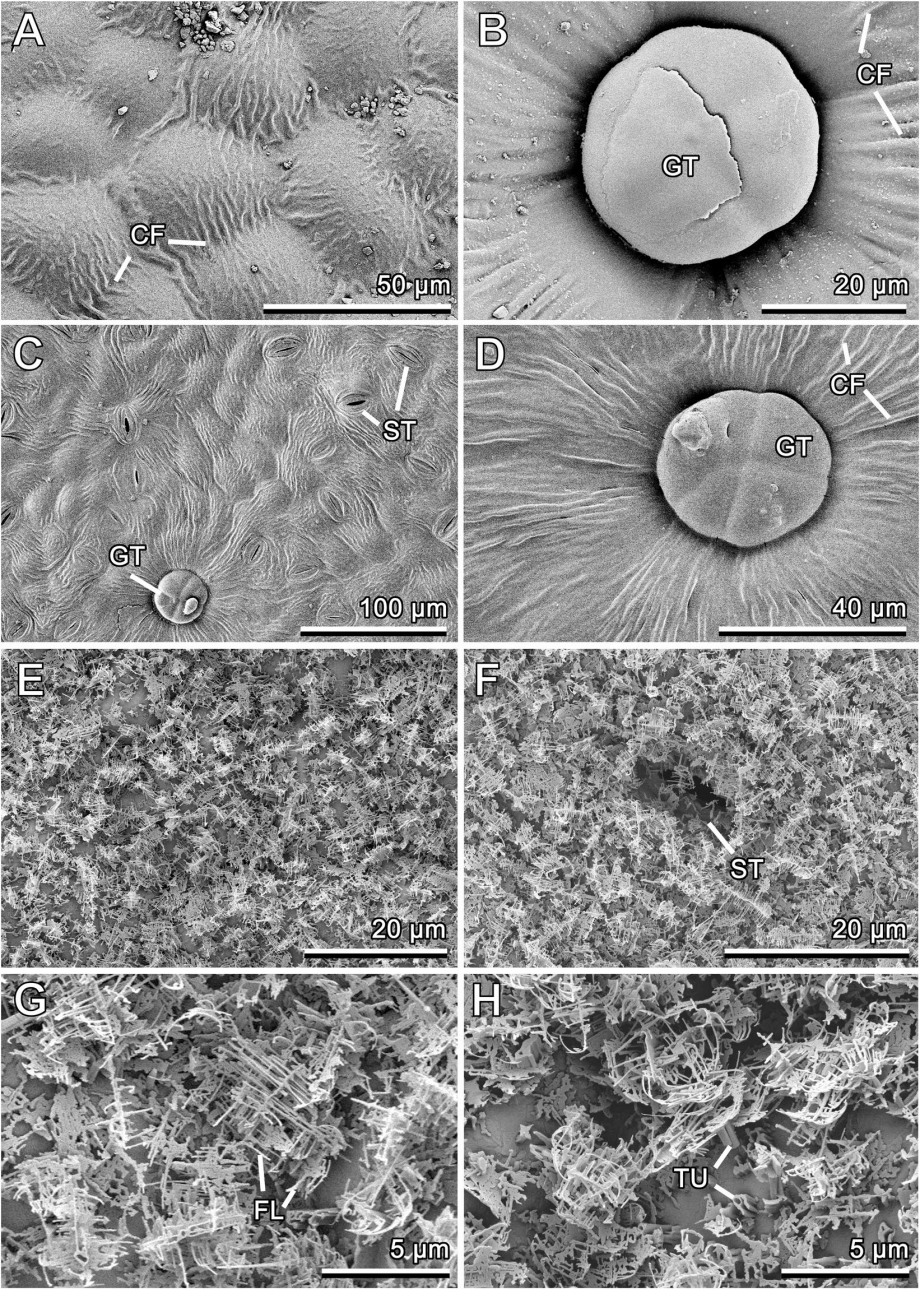
Cryo-SEM micrographs of the adaxial (**A**,**B**) and abaxial (**C**,**D**) leaf surfaces in *Syringa vulgaris* and of the adaxial (**E**,**G**,**H**) and abaxial (**F**) leaf surfaces in *Brassica oleracea*. (**A**) Cuticular folds running between neighboring cells. (**B**,**D**) Glandular trichomes and cuticular folds running radially. (**C**) General view showing different surface features. CF, cuticular fold; FL, wax filament; GT, glandular trichome; ST, stoma; TU, wax tubule.

Both *Syringa vulgaris* leaf surfaces being somewhat uneven due to slightly convex epidermal cells shapes bear scattered peltate glandular trichomes and are richly microstructured with cuticular folds (Fig. 4A,C). These rather small (head diameter: 32.27 ± 5.07 μm, N = 12) globular trichomes with at least four glandular secretory cells (the exact number was hardly distinguishable because of the subcuticular storage cavities filled with the secretion on cells tops) are located in surface depressions (Fig. 4B,D). They are evenly distributed over the surface, with the density ca. 9–11 trichomes per mm^2^. Numerous fine cuticular folds (width: 1.30 ± 0.55 μm, N = 20) densely cover the surface. On the adaxial side, they are especially pronounced at the areas between neighboring cells (Fig. 4A). The folds are much longer in the vicinity of peltate trichomes (both leaf sides) and some stomata (abaxial side), where they run radially (Fig. 4B–D). On the abaxial side additionally, they are much longer and run parallel to each other along the leaf veins. Although stomata are present, they are almost not seen on the adaxial side, whereas they are distinct and in a great number (density: ca. 140 mm^−2^) on the abaxial leaf surface (Fig. 4C).

Both sides of the *Brassica oleracea* leaf show rather similar surface micromorphology (Fig. 4E,F). Very prominent 3D epicuticular wax that covers the surface completely and uniformly is composed of round/angular tubules (Fig. 4H) bearing characteristic dendrite-like branches on their tops (Fig. 4G,H). The tubules protruding more or less perpendicularly from surface are almost entirely overlaid by the branches oriented nearly parallel to the surface. The branches are formed by thin filaments of varying length (length: 1.98 ± 0.85 μm, N = 13; diameter: 0.06 ± 0.02 μm, N = 10), which are partially fused to different degrees (Fig. 4G). There is a certain difference in the density of the 3D wax coverage between the leaf sides: it is nearly two-fold lower on the adaxial leaf side, if compared to that on the abaxial side (ca. 10 and 20 wax projections per 100 μm^2^, respectively) (compare Fig. 4E and 4F). Stomata (Fig. 4F) are present on both sides, but occur there in different numbers: they are distributed more sparsely (density: ca. 43 mm^−2^) on the adaxial side and much more densely (density: ca. 263 mm^−2^) on the abaxial side.

**Figure 5:**
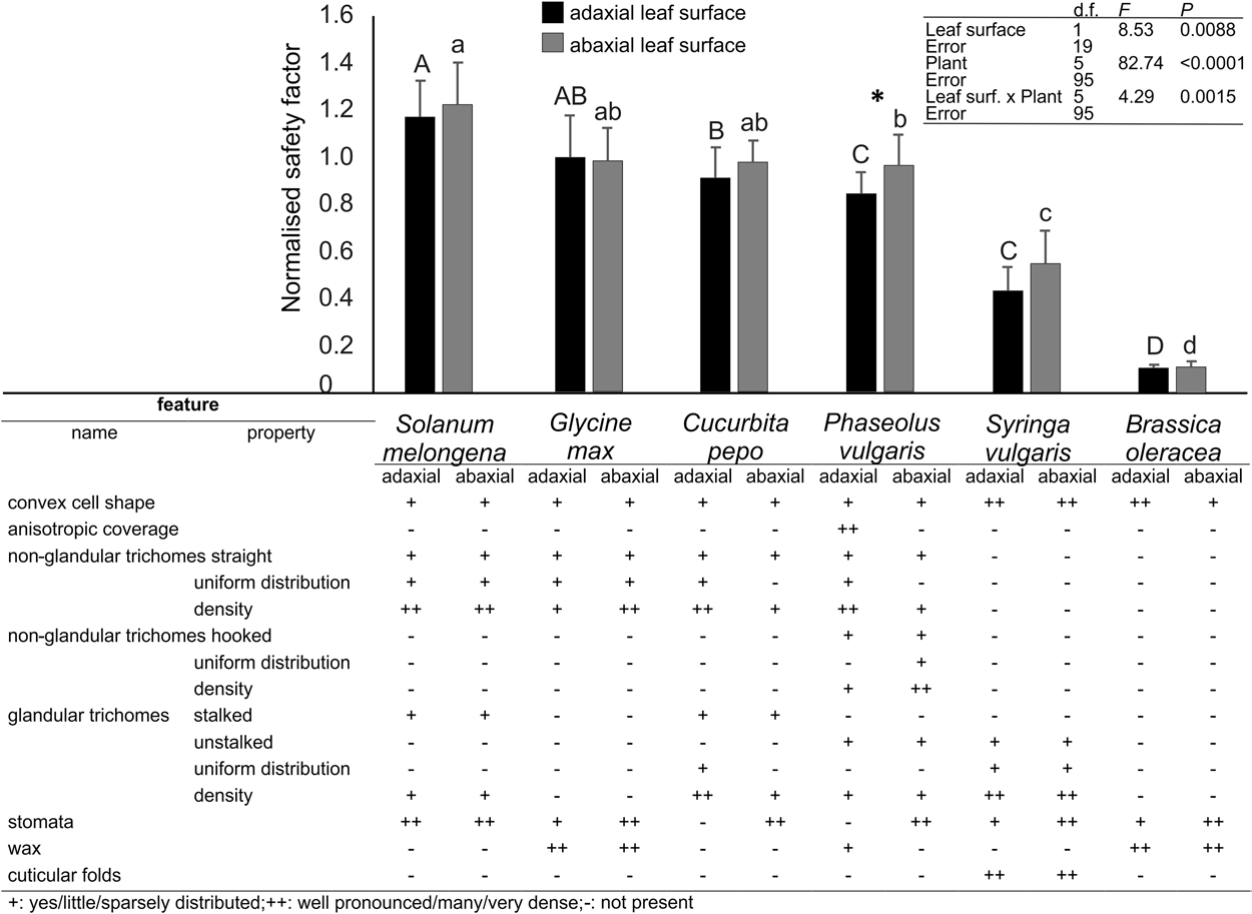
Normalised safety factor generated by *Nezara viridula* on adaxial and abaxial leaf surfaces of different plant hosts with a summary of the most relevant features of adaxial and abaxial leaf surfaces of the tested plants. Bars indicate the means ± s.e.m. In comparison among data on adaxial and among abaxial leaf surface, column with different upper case letters and lower case letters, respectively, are significantly different at P < 0.05. Couples of columns with an asterisk are significantly different at P < 0.05, Fisher LSD post-hoc test. Table inset shows the statistical parameters of two-way repeated measures ANOVA.

The most relevant surface features of leaves in different plants examined here are summarized in Fig. 5.

### Attachment ability on different host plant species

In the traction force experiments, aiming to evaluate the attachment ability of *N*. *viridula* on different host plant species, the normalized safety factor varied significantly depending on the leaf surface, as well as on the plant species. The interaction between leaf surface and plant species was also statistically significant (Fig. 5). *N*. *viridula* realized the same attachment on both sides of each plant leaf, except for *P*. *vulgaris*, where the normalized safety factor was higher on the abaxial than on the adaxial surface. Among different tested adaxial leaf surfaces, the higher normalized safety factor was recorded on *S*. *melongena* and *G*. *max*, followed by *C*. *pepo*. The lower normalized safety factor was recorded on *B*. *oleracea*, while it was intermediate on *P*. *vulgaris* and *S*. *vulgaris* without any difference between them. Among the different tested abaxial leaf surfaces, the higher normalized safety factor was recorded on *S*. *melongena*, *G*. *max* and *C*. *pepo* followed by *P*. *vulgaris*. The lower normalized safety factor was recorded on *B*. *oleracea*, while it was intermediate on *S*. *vulgaris* (Fig. 5).

### Damage to the insect induced by walking on different leaf surfaces

**Figure 6:**
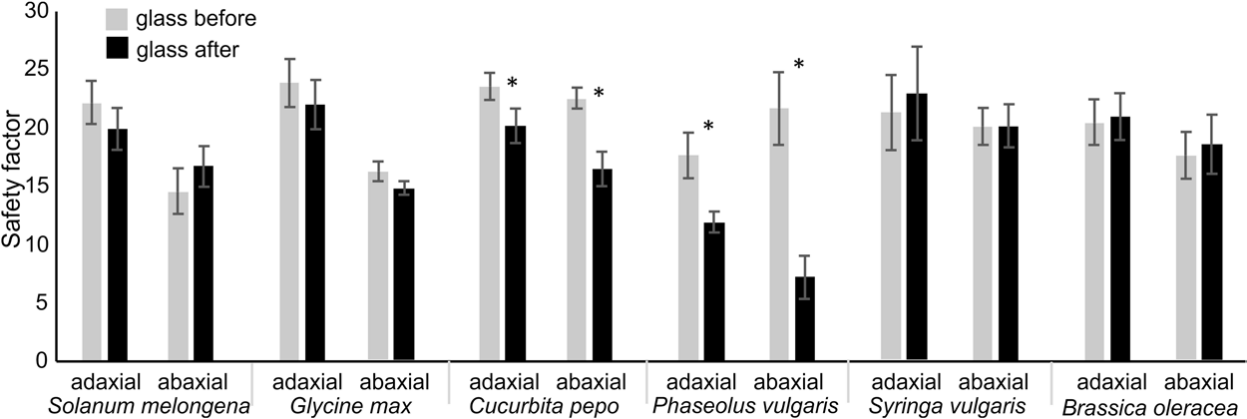
Safety factor (friction force divided by the insect weight) obtained with *Nezara viridula* on glass before and after walking on adaxial and abaxial leaf surfaces of different tested plant species. Bars indicate the means ± s.e.m. Columns with asterisk are significantly different at P < 0.05, Student’s *t*-test for dependent samples.

In the traction force experiments, measuring the friction force produced by insects on glass before and after walking on each side of different tested plant species, aiming to evaluate a possible damage of attachment devices, no significant difference between the two glass surfaces was recorded for both adaxial and abaxial surfaces in *S*. *melongena* (adax.: t = 1.61; d.f. = 5; p = 0.17. abax.: t = 1.43; d.f. = 5; p = 0.21), *G*. *max* (adax.: t = 1.87; d.f. = 5; p = 0.12. abax.: t = 1.63; d.f. = 5; p = 0.16), *S*. *vulgaris* (adax.: t = 1.05; d.f. = 5; p = 0.34. abax.: t = 0.14; d.f. = 5; p = 0.89) and *B*. *oleracea* (adax.: t = 0.37; d.f. = 5; p = 0.73. abax.: t = 1.18; d.f. = 5; p = 0.29). A significant reduction of safety factor was recorded for both leaf sides in *C*. *pepo* (adax.: t = 4.41; d.f. = 5; p = 0.007. abax.: t = 4.09; d.f. = 5; p = 0.010) and in *P*. *vulgaris* (adax.: t = 2.85; d.f. = 5; p = 0.036. abax.: t = 3.82; d.f. = 5; p = 0.012), where the reduction was particularly evident (Fig. 6).

### Damage to insects by *P. vulgaris* leaves and insect ability to recover after 24 h

**Figure 7:**
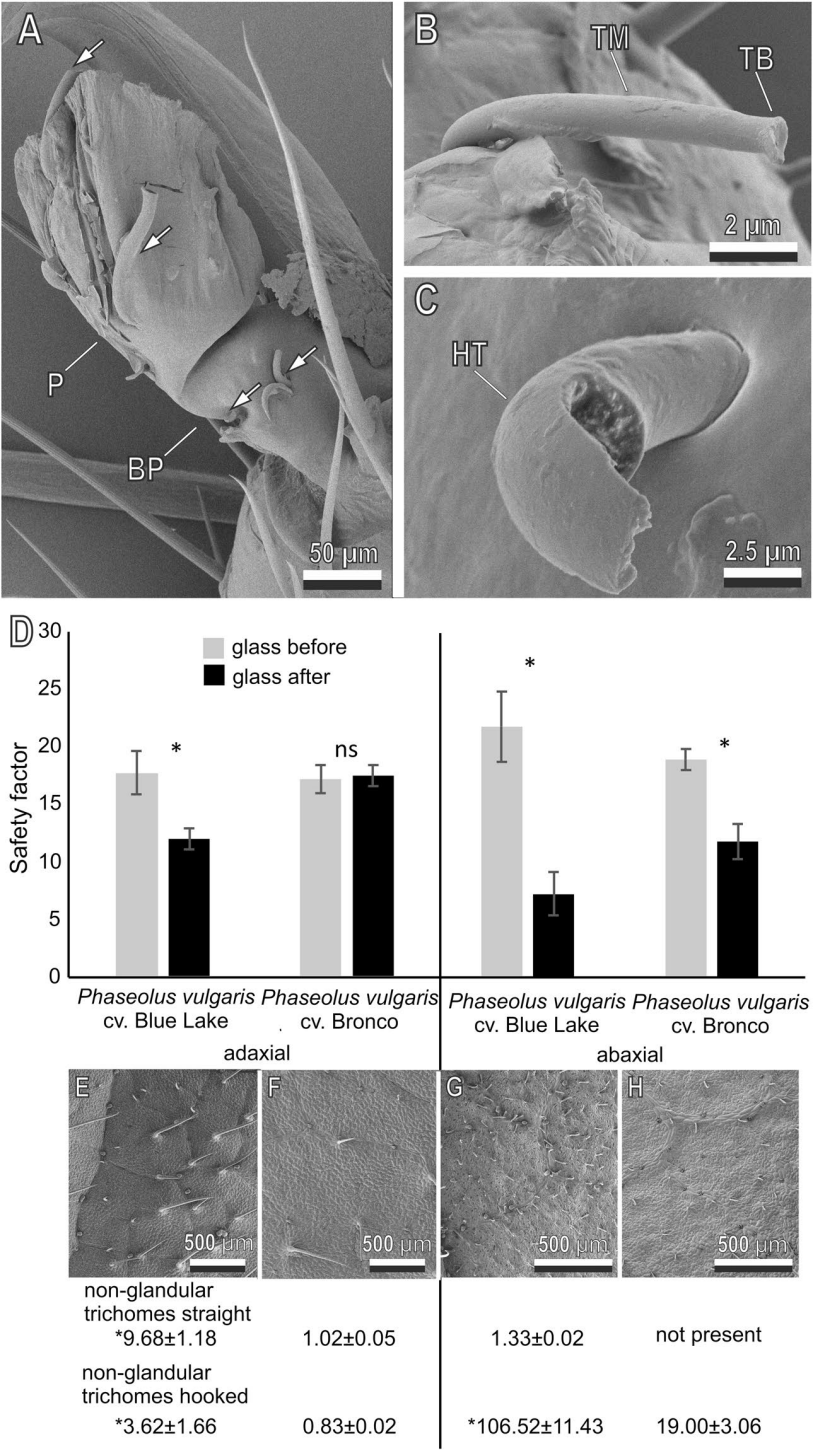
Damage to *Nezara viridula* by the presence of hooked trichomes in *Phaseolus vulgaris* leaves. Cryo-SEM micrographs of damaged pulvilli (**A**–**C**). **A** Pulvillus (P) and basipulvillus (BP) impaled by several hooked trichomes (arrows). (**B**) Hooked trichomes penetrated in the pulvillus broken at the solid base (TB), TM, middle part. (**C**) Hooked trichomes penetrated in the pulvillus broken along the stable solid hooked part (HT). (**D**) Safety factor (friction force divided by the insect weight) obtained with *Nezara viridula* on glass before and after walking on adaxial and abaxial leaf surfaces of the cv. Blue lake and cv. Bronco of *Phaseolus vulgaris*. Bars indicate the means ± s.e.m. Columns with an asterisk are significantly different at P < 0.05, Student’s *t*-test for dependent samples. Cryo-SEM micrographs of the adaxial (**E**,**F**) and abaxial (**G**,**H**) leaf surfaces and density (mean ± s.e.m. of number mm^−2^) of non-glandular straight and hooked trichomes of the two cultivars of *P*. *vulgaris*. For each leaf surface, trichome densities with an asterisk are significantly different at P < 0.05, Student’s *t*-test for independent samples.

Cryo-SEM investigations performed on insects after walking on the abaxial leaf surfaces of *P*. *vulgaris* revealed that the pulvilli of *N*. *viridula* are damaged by the presence of hooked trichomes penetrated inside the pulvilli (Fig. 7A–C). These hooked trichomes after their penetration inside the pulvilli can be broken by the insect during pulling at the level of their solid base (Fig. 7B) or along the stable solid hooked part (Fig. 7C).

Cryo-SEM investigations on the leaf surfaces of two cultivars of *P*. *vulgaris* with different morphological features, reveal that the adaxial surface of the cv. Bronco bears less non-glandular straight trichomes (t = 4.34; d.f. = 2; p = 0.049) and very few hooked trichomes (t = 3.27; d.f. = 3; p = 0.047) compared to the cv. Blue lake (Fig. 7E,F), while the abaxial leaf surface is characterized by the absence of non-glandular straight trichomes and by the presence of hooked trichomes with a lower density (t = 3.62; d.f. = 3; p = 0.036), if compared to the cv. Blue lake (Fig. 7G,H). In the traction force experiments on *P*. *vulgaris* leaves of the two different cultivars, the normalized safety factor recorded on the adaxial leaf surface was higher (t = 2.23; d.f. = 28; p = 0.034) on the cv. Bronco (1.18 ± 0.11) than on the cv. Blue lake (0.84 ± 0.09), while the normalized safety factor, recorded on the abaxial leaf surface, was not statistically different (t = 0.31; d.f. = 33; p = 0.75) on the two cultivars (cv. Bronco: 0.91 ± 0.06; cv. Blue lake: 0.96 ± 0.13). The safety factor, recorded in insects on glass before and after walking on each side, was compared in the two cultivars of *P*. *vulgaris*, in order to verify the role of plant morphological features in producing damage to the insect attachment devices. The safety factor on glass after walking on the adaxial leaf surface was significantly lower than that on glass before in the cv. Blue lake (t = 2.90; d.f. = 5; p = 0.034), but not in the cv. Bronco (t = 0.28; d.f. = 9; p = 0.78). After walking on the abaxial leaf surface, the safety factor on glass was lower than on glass before for both cultivars (cv. Blue lake: t = 4.64; d.f. = 5; p = 0.006; cv. Bronco: t = 6.03; d.f. = 14; p < 0.0001) (Fig. 7D).

**Figure 8:**
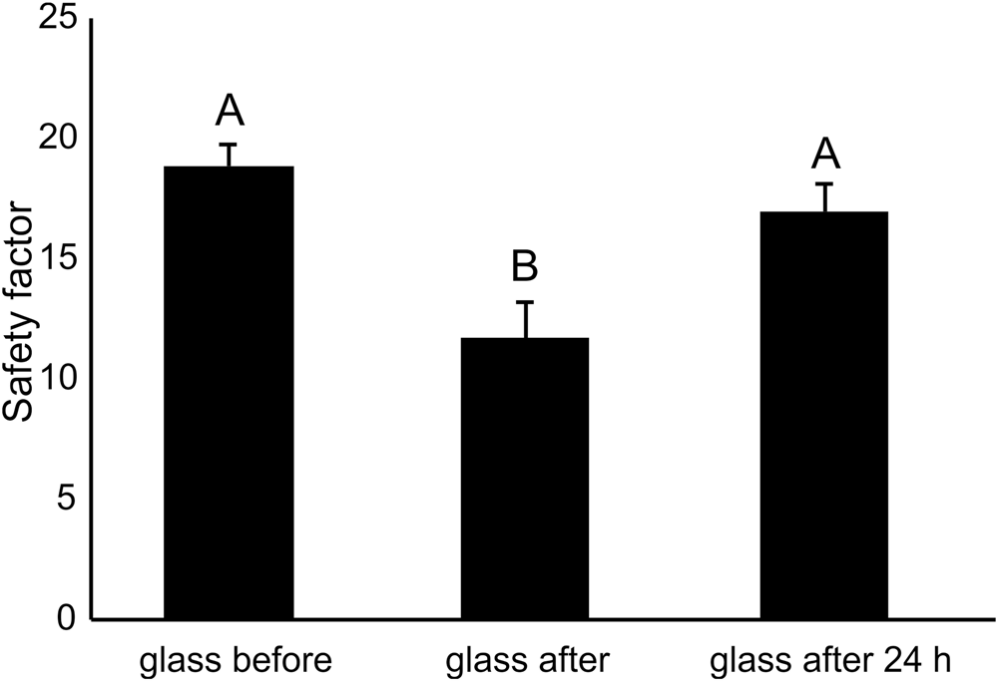
Safety factor (friction force divided by the insect weight) obtained with *Nezara viridula* on glass before, glass after and glass after 24 h walking on the abaxial leaf surfaces of the cv. Bronco of *Phaseolus vulgaris*. Bars indicate the means ± s.e.m. Columns with different letters are significantly different at P < 0.05. One-way repeated measures ANOVA, Fisher LSD post-hoc test.

The attachment ability was recorded in insects on glass (1) before, (2) just after and (3) 24 h after walking on abaxial leaf side of the cv. Bronco, in order to verify the possible recovery ability of the insects. The safety factor was significantly lower on glass just after walking on the leaf surface, while no difference was recorded between the sample “before” and the sample “24 h after” (F = 17.11; d.f. = 14, 2, 28; p < 0.0001) (Fig. 8).

## Discussion

The data reported in the present investigation, aiming to evaluate the attachment ability of *N*. *viridula* at the adult stage on different host plant species, reveal that *N*. *viridula* realizes the same attachment on both sides of each plant, except for *P*. *vulgaris*. The best adhesion is realized on *S*. *melongena*. This means that the dense pubescence, formed by non-glandular stellate trichomes with vertical arms and accumbent side arms present on both leave surfaces of this plant species, does not reduce the insect attachment ability but, on the contrary, is probably used by insect claws to improve attachment during pulling. Notwithstanding one of the main function of plant trichomes is to contribute to the plant defense mechanism against herbivores (see review by^2^), for some insects on some plants, it was reported that trichomes provide an additional “foothold” and thus may promote insect attachment to a plant^4,8,29,30^. Attachment tests on the chrysomelid beetle *Phaedon cochleariae* on different mutants of *Brassica* sp. revealed that trichomes may provide clinging sites for the tarsal claws^29^. Attachment ability of the beetle *Chrisolina fastuosa* to hairy plant substrata with trichomes of different sizes showed that the beetles attached well to all these surfaces and the size and density of trichomes seemed to have no significant effect on attachment^30^. The omnivorous mirid bug *Dicyphus errans* lives on pubescent plants and shows morphological (elongated curved claws, long and slender legs) and behavioural (specialised locomotion) adaptations to hairy plant substrates. The traction force of *D*. *errans* was measured on different hairy plants among which *S*. *melongena* and a significant positive correlation between the force and both the trichome length and diameter was found^8^.

A good adhesion has been realised by *N*. *viridula* also on *G*. *max* and *C*. *pepo* leaves. Both sides of *G*. *max* leaves are characterised by the presence of long and thin non-grandular trichomes and by a very uniform, regular and dense three-dimensional epicuticular wax coverage composed of one layer of irregular platelets. Probably, the ability of *N*. *viridula* to attach to this plant is guaranteed by the reduced thickness of the wax layer, if it is compared to other plant species. Indeed, not only the presence of cristalline wax, but also the crystal length and density of the epicuticular wax coverage may effect insect attachment, as observed for the beetle *Cryptolaemus montrouzieri* (hairy pads) moving on the leaflets of the *Pisum sativum* plants with wild-type surface waxes and with reduced surface waxes, caused by a mutation *wel* (wax eliminator)^31^. Moreover in *G*. *max*, the presence of long trichomes, as above reported, can provide clinging sites for the claws. This last property can be attributed also to the non-branched, cone-shaped multicellular non-glandular trichomes with sharp tips featuring the typical pubescence of the leaves of *C*. *pepo*.

Adhesion of *N*. *viridula* to *P*. *vulgaris* is higher on the abaxial side of the leaf, if compared with the adaxial side. Indeed, the two sides of the leaf show different features due to the different distribution of the two kinds of non-glandular trichomes typical of this plant species. The adaxial side is characterized by a great number of non-glandular trichomes with very sharp tip protruding at very shallow angles and pointed to one preferred direction, responsible for a regular and dense anisotropic coverage on the surface. The abaxial side is characterized by prevalence of a second type of non-glandular trichomes with characteristic hook-shaped structures having sharp tips pointed to different directions. These hooked trichomes responsible for the ability of bean leaves to entrap insects^28,32–36^ are more abundant on the abaxial side. In any case, they are probably useful for *N*. *viridula* to attach to the surface, when pulling parallel or perpendicular to the leaf surface. The lower traction force, recorded on the adaxial leaf side, is probably due to the surface anisotropy that does not facilitate claw interlocking, as previously reported^37^. The role of anisotropy in the decrease of *N*. *viridula* attachment ability is confirmed by the higher normalized safety factor recorded on the cv. Bronco (characterized by very few non-glandular straight trichomes and consequently by absence of anisotropy), if compared to the cv. Blue lake.

On *S*. *vulgaris*, we observed a lower traction force, if compared to the above discussed plant species, on both leaf sides (similar only to the adaxial side of *P*. *vulgaris*). This reduction in attachment ability can be due to the numerous fine cuticular folds densely covering the adaxial and abaxial surface of this plant species. Based on a comparative SEM study of the functional surfaces in carnivorous plants and kettle trap flowers, the folds found in 11 species were regarded as structures preventing adhesion of insect pads due to contact area reduction caused by surface micro-roughness created by folds^38^. The effect due to the cuticular folds has been studied in detail with traction experiments in the Colorado potato beetle on five plant surfaces with cuticular folds of different magnitude^39^. These cuticular folds reduce strongly (mainly the leaf surfaces with medium cuticular folds) the beetle adhesion in comparison to smooth plant surfaces without cuticular folds, thus leading to the hypothesis that cuticular folds decrease insect adhesion mainly due to a critical microroughness, reducing the real contact area between the surface and the insect’s adhesive devices (pulvilli and hairy pads).

In *N*. *viridula*, the reduction of adhesion on microrough surfaces has been proved in traction force experiments on artificial substrates with different roughness^13,22^. On the other hand, keeping in mind that the diameter of claw tips of *N*. *viridula* is about 8 µm^13^, the claws probably are not able to interlock with the cuticular folds which in *S*. *vulgaris* have spacing between them of about 2 µm^5,40^. According to this hypothesis, demonstrated in different insects, cuticular folds may reduce insect attachment ability similarly to the presence of wax crystals (roughness hypothesis according to^30^)^29,31,39,41,42^. Plant surfaces with 3D epicuticular waxes have been shown to strongly reduce insect adhesion^29,31,37,41,43–46^. The very prominent and complex 3D epicuticular wax that covers both sides of the leaf surface of *B*. *oleracea* induces a strong reduction in traction force of *N*. *viridula*. In *S*. *vulgaris*, the impact on the reduction in traction force due to the cuticular folds is not so high as that due to the waxes in *B*. *oleracea*, probably because of the compensation effect of the convex cell shape of *S*. *vulgaris*. Indeed traction forces on plant surfaces with convex cells are about 1.5 times as high as on plant surfaces possessing tabular epidermal cells, as shown by^42^.

As far as the presence of glandular trichomes is concerned, stalked ones have been observed on both adaxial and abaxial surfaces of *S*. *melongena* and *C*. *pepo*, while non stalked glandular trichomes have been observed on both leaf sides of both *P*. *vulgaris* and *S*. *vulgaris*. Some experimental studies demonstrated a decrease in insect attachment ability to plant surfaces bearing glandular trichomes^30,47,48^. In our investigation, their presence seems to have no detrimental effect on the traction force of *N*. *viridula* presumably because of the large overall size of the tested insects.

In the traction force experiments, friction forces of insects measured on glass before and after walking on each leaf side of different tested plant species aimed to evaluate a possible damage to insect attachment devices. A significant reduction of safety factor on the repeated glass test was recorded only for both leaf sides of *C*. *pepo* and *P*. *vulgaris*. As far as *C*. *pepo* is concerned, to the best of our knowledge, this is the first report of damage to the walking ability of an insect by this plant. We presume that the developed multicellular, non-branched, cone-shaped, trichomes with sharp tips could be involved in producing some damage to the insect, but further experimental investigations are necessary to clarify this point. The decrease in the attachment ability of *N*. *viridula* after walking on bean leaves has been highlighted in a recent investigation by^18^. In this paper, this reduction was explained by the contamination of pulvilli and hairy pad by epicuticular wax crystals. The cultivar of *P*. *vulgaris* tested by us, showed very low crystalline wax coverage (probably less that that tested by^18^) but, in any case, the explanation by^18^ is not in agreement with the data shown in the present investigation, because in our experiments *N*. *viridula* attachment devices were not damaged by the presence of waxes (we did not observe any reduction in traction force of *N*. *viridula* after walking on *G*. *max* and *B*. *oleracea*, which show a high amount of epicuticular waxes, if compared with *P*. *vulgaris*). Moreover, our cryo-SEM investigation on *N*. *viridula* pulvilli after pulling on the abaxial leaf surface of *P*. *vulgaris*, which is rich of non-glandular trichomes with characteristic hook-shaped structures having sharp tips pointed to different directions, reveals clearly that these trichomes are able to penetrate deeply inside the ventral surface of pulvilli and are able to break at some loading conditions. The ability of the hook-shaped trichomes of *P*. *vulgaris* to entrap small insects, such as leafhoppers, aphids and bed bugs by impaling them is well-known^28,32–36^, but, as far as we know, this is a first report of damage by *P*. *vulgaris* leaves to an insect of such a big size, as *N*. *viridula*. The reduction in traction force of *N*. *viridula* due to the damage by the hooked trichomes is further confirmed by the different traction force of *N*. *viridula* on glass after walking on two cultivars of *P*. *vulgaris* with different morphological features. Indeed, the safety factor on the second glass, after walking on the adaxial leaf surface, was significantly lower than the safety factor on glass before in the cv. Blue lake, which bears more hooked trichomes, but not in the cv. Bronco, which bears only very few hooked trichomes.

In our experiments to verify the recovery ability of *N*. *viridula* 24 h after walking on the abaxial leaf side of the cv. Bronco of *P*. *vulgaris*, the safety factor was significantly lower on glass just after walking on the leaf surface, while no difference was recorded in the safety factor between glass before and 24 h after, thus, showing that after 24 h, pulvilli of *N*. *viridula* were able to attach again to the tested surface. During pulling, adult stink bugs may be damaged by hooked trichomes but considering that they can fly and thus move to alternative host plants, they can easily recover. The impact of hooked trichomes on nymphs could be higher considering that they cannot fly and it would be interesting to evaluate this hypothesis in future bioassays.

Strong friction forces produced by *N*. *viridula* on plant leaves, characterized by different combinations of textures and wettability properties, is in agreement with the great adaptability of this species as it is confirmed by its polyphagy and its worldwide distribution^49^. The high ability of this insect pest to attach to a variety of plant surfaces could be allowed by the presence of different types of tarsal pads (smooth pulvilli and hairy adhesive pads) shown for *N*. *viridula*^21^. In any case, recent investigation on the contribution of different tarsal attachment devices to the overall attachment ability of this insect on flat artificial substrates of different roughness revealed a great involvement of pulvilli in insect attachment on all the tested surfaces, while hairy pad had its main role in producing friction forces only on smooth surfaces, on surfaces with intermediate roughness and on hydrophobic substrates under water^22^. Examining the contact area of attachment devices of *N*. *viridula*, the involvement of pulvilli in adhesion is higher than that of hairy pad and is not different both during pulling and inverted climbing^22^. For this reason, we can assume a similar role of plant surface morphology in increasing or reducing the insect attachment ability during pulling (tested in the present investigation) or inverted walking, a behaviour frequently observed in *N*. *viridula* adult females laying eggs on the abaxial leaf surface.

We believe that the present study increases our knowledge about mechanical interactions of this species with different host plant species and can help to develop strategies useful to control this insect pest. Such eco-friendly crop protection strategies could be achieved due to the decreasing of attachment ability of insect pest through the genetic modification of crop cultivars characterised by higher amount of crystalline waxes, cuticular folds or entrapping trichomes.

## Material and Methods

### Insects

*N*. *viridula* bugs were collected in the field in July 2016 close to Foligno (Perugia, Umbria region, Italy) and reared in a controlled condition chamber (14 h photophase, temperature of 25 ± 1 °C; RH of 70 ± 10%), inside clear plastic food containers (300 mm × 195 mm × 125 mm) with 5 cm diameter mesh-covered holes. All stages were fed with seeds, fruits and vegetative parts of their preferred food plants. In particular, sunflower seeds (*Helianthus annus* L.) and French beans (*Phaseolus vulgaris* L.) were used to feed the insects. In consideration that the behavioural investigations on the attachment ability of males and females of *N*. *viridula* on hydrophilic and hydrophobic artificial surfaces and on the adaxial and abaxial leaf surfaces of the model plant species *Vicia faba*^13^ did not reveal any difference between the two sexes, the present investigation has been performed only on adult males.

### Plants

Six common garden plant species chosen among the host plants of *N*. *viridula*^49^ with leaves having different surface texture were used in this study: eggplant *Solanum melongena* L. (Solanaceae), soybean *Glycine max* (L.) Merr (Fabaceae), zucchini *Cucurbita pepo* L. subsp. *pepo* convar. *giromontiina* (Cucurbitaceae), common bean *Phaseolus vulgaris* L. (Fabaceae), common lilac *Syringa vulgaris* L. (Oleaceae), and cabbage *Brassica oleracea* L. var. *capitata* (Brassicaceae).

For both behavioural and morphological investigations all the plants have been used at the stage of 6–8 true leaves except for *S*. *vulgaris* for which leaves from a three years old shrub in the leaf stage have been used.

### Cryo-scanning electron microscopy (Cryo-SEM)

The shock-frozen samples of abaxial and adaxial leaf surfaces of the selected plant species and the tarsal attachment devices of *N*. *viridula* insects were studied in a scanning electron microscope (SEM) Hitachi S-4800 (Hitachi High-Technologies Corp., Tokyo, Japan) equipped with a Gatan ALTO 2500 cryo-preparation system (Gatan Inc., Abingdon, UK). For details of sample preparation and mounting for cryo-SEM, see^41^. Cryo-SEM is useful where preservation of the natural ‘life-like’ morphology of cells and tissues is desired, in consideration that cryofixation is rapid and immobilises processes at a much faster rate than chemical fixation^50^. Additionally, it allows visualization of fluids, which might be crucial in the case of insect attachment devices. Whole mounts of small leaf pieces and insect tarsi were sputter-coated in frozen conditions with gold-palladium (thickness 10 nm) and examined at 3 kV acceleration voltage and temperature of −120 °C in the microscope. In each plant species, we examined two-three areas of both sides of two-three mature leaf from the plants used for experiments with bugs. These leaves were at the same developmental stage as those used in the experiments.

### Force measurements

The tests were performed using a traction force experiments set up. Prior to the force measurements, male adults of *N*. *viridula* were weighted on a micro-balance (Mettler Toledo AG 204 Delta Range, Greifensee, Switzerland). Experimental insects were anaesthetized with carbon dioxide for 60 s, were made incapable of flying by carefully gluing their forewings together with a small droplet of melted wax, and one end of a 15–20 cm long human hair was fixed with a droplet of molten wax on their pronotum. Before starting the experiments, insects were left to recover for 30 min.

All the experiments were performed during the daytime at 22 ± 1 °C and 45 ± 5% relative humidity.

The traction force experiments set up consisted of a force sensor FORT-10 (10 g capacity; World Precision Instruments Inc., Sarasota, FL, USA) connected to a force transducer MP 100 (Biopac Systems Ltd, Goleta, CA, USA)^51^. Data were recorded using AcqKnowledge 3.7.0 software (Biopac Systems Ltd, Goleta, CA, USA). The insect was attached to the force sensor by means of the hair glued to its pronotum and was allowed to move on the substrate and to be tested in a direction perpendicular to the force sensor. The force generated by the insect walking horizontally on the test substrates was measured. Force–time curves were used to estimate the maximal pulling force produced by running insects (friction). Three sets of experiments have been performed:

#### Attachment ability on different host plant species

Two leaves of each plant species have been cut from the plant and attached (one leaf on its abaxial side and the other on the adaxial side) with double-sided tape to a horizontal glass plate, trying to keep the leaves very flat on the glass plate. The leaves were changed after every three tested insects to avoid dehydration of leaves. Each insect walked first on glass, and then from the proximal to the distal portion of the two leaf sides of each plant species, presented in random order. Between one leaf surface and the other, the insect walked for some seconds on the filter paper to promote leg cleaning. The maximal friction force produced by insects on different test surfaces was estimated for each individual run. In total, 20 males were tested and the obtained maximal forces were averaged for each single surface tested.

#### Damage of insects induced by walking on the different leaf surfaces

Two leaves (abaxial and adaxial side) of each plant species have been cut from the plant and attached to a horizontal glass plate as above described, changing them every three tested insects to avoid leaf dehydration. Each insect walked first on glass, and then from the proximal to the distal portion of a leaf side of each plant species, and finally again on glass. The maximal friction force produced by insects on glass before and after walking on each side of each tested plant species was estimated and compared. In total, six males for each leaf surface were tested.

#### Damage of insects by *P*. *vulgaris* leaves and their ability to recover after 24 h

Two leaves (abaxial and adaxial sides) of *P*. *vulgaris* of two different cultivars (cv. Blue lake and cv. Bronco) with different morphological features have been cut from the plant and attached to a horizontal glass plate as described above, changing them after every three tested insects to avoid leaf dehydration. Each insect walked first on glass, and then from the proximal to the distal portion of a leaf side, and finally again on glass. The maximal friction force produced by insects on glass before and after walking on the plant was estimated and compared. In total, 6–15 males were tested on each leaf side of both cultivars. In the case of the abaxial leaf surface of the cv. Bronco, the recovery ability of the insects has been evaluated by testing their attachment ability on glass after 24 h. During the 24 h each insect has been kept in a plastic Petri dish with some food. The maximal friction force produced by insects on glass (1) before, (2) just after and (3) after 24 h of recovery was estimated and compared. In total, 15 males were tested.

### Statistical analysis

The maximal friction force produced by insects on different plant surfaces was divided by the body mass, to obtain the safety factor. In the experiment, to evaluate the attachment ability on different host plant species, the safety factor was normalized as percentage of the maximal force, produced on glass, to reduce the variability caused by individual insects. Obtained data were analyzed with 2-way repeated measures ANOVA^52^ considering the leaf surfaces and the plants as factors. F tests were used to assess the significance of the factors and the significance of their interactions. For significant factors, the Fisher LSD test was used as post-hoc test^52^. To evaluate the damage to the insect induced by walking on leaf surfaces of different plant species and especially on the two *P*. *vulgaris* cultivars, the safety factor recorded on the glass before and on the glass after walking on each leaf surface was compared using the Student’s *t*-test for dependent samples. In the evaluation of the recovery ability of the insects, the safety factor recorded on glass (1) before, (2) just after and (3) 24 h after walking on the abaxial leaf side of bean plant were compared using a repeated measures ANOVA, and the Fisher LSD test was used as post-hoc test. The trichome densities of the two cultivars of *P*. *vulgaris* were compared for each leaf surface using the Student’s *t*-test for independent samples. The same test was used to compare the normalized safety factor recorded on the leaf surface of *P*. *vulgaris* cultivars^52^. Before the analysis, all the data were subjected to Box–Cox transformations, in order to reduce data heteroscedasticity^53^.

### Data availability

The datasets generated during and/or analysed during the current study are available from the corresponding author on reasonable request.

## Electronic supplementary material

Supplementary Figure
