# Supplementary material for: Attachment ability of the polyphagous bug Nezara viridula (Heteroptera: Pentatomidae) to different host plant surfaces
Source: Sci Rep. 2018 Jul 20;8:10975. doi: 10.1038/s41598-018-29175-2 (PMC6054675; doi:10.1038/s41598-018-29175-2)
Supplement: Supplementary file 1 — Supplementary Figure [file 41598_2018_29175_MOESM1_ESM.pdf]

Attachment ability of the polyphagous bug *Nezara viridula* (Heteroptera: Pentatomidae) to different host plant surfaces

Gianandrea Salerno, Manuela Rebora, Elena Gorb, Stanislav Gorb

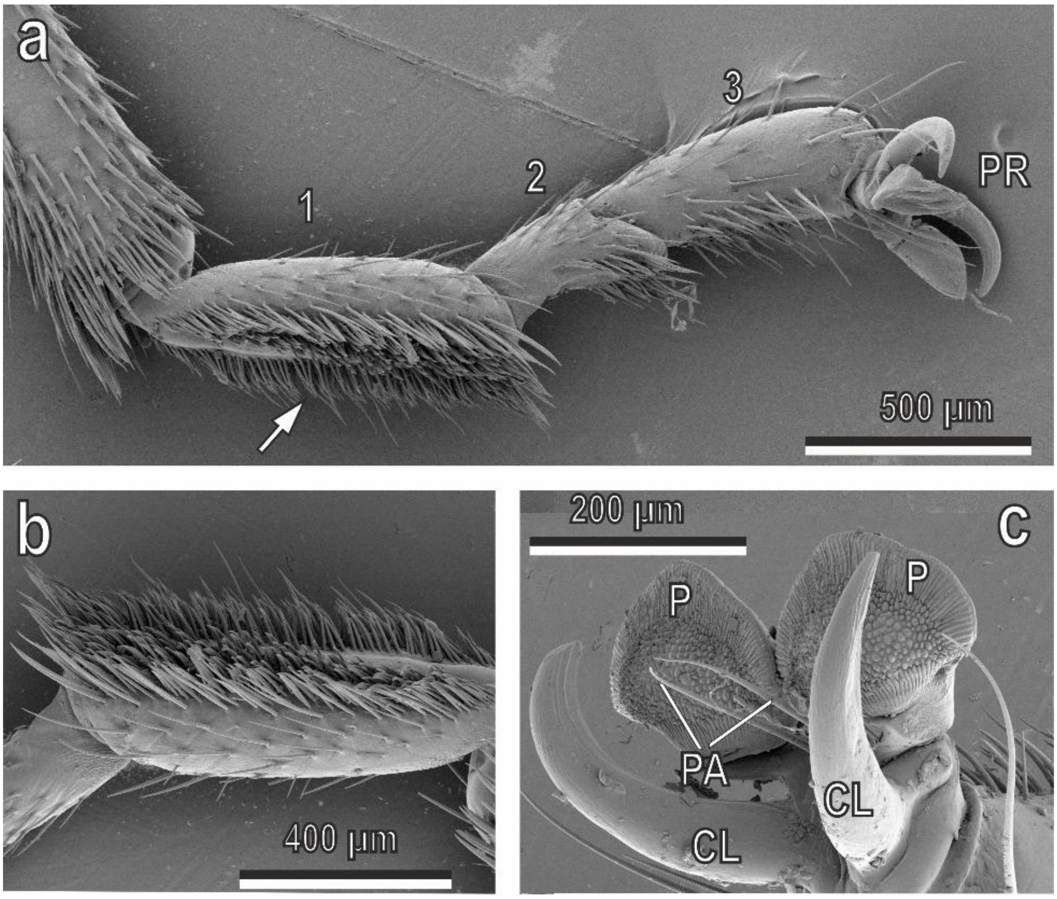

Supplementary Fig. S1 online. Tarsus of *Nezara viridula* visualized under cryo - SEM. **a** Lateral view of the three tarsal segments (1-3) and of the pretarsus (PR). Note the ventral adhesive setae at the basitarsus (arrow). **b** Ventral view of the basitarsus showing the two rows of setae of the hairy adhesive pad. **c** Dorsal view of the pretarsus showing two curved claws (CL), two pulvilli (P) and two paraempodia (PA).
